# FTSJ1 regulates tRNA 2ʹ-O-methyladenosine modification and suppresses the malignancy of NSCLC via inhibiting DRAM1 expression

**DOI:** 10.1038/s41419-020-2525-x

**Published:** 2020-05-11

**Authors:** Qihan He, Lin Yang, Kaiping Gao, Peikun Ding, Qianqian Chen, Juan Xiong, Wenhan Yang, Yi Song, Liang Wang, Yejun Wang, Lijuan Ling, Weiming Wu, Jisong Yan, Peng Zou, Yuhuan Chen, Rihong Zhai

**Affiliations:** 1 School of Public Health, Guangdong Key Laboratory for Genome Stability & Disease Prevention, Carson Cancer Center, Shenzhen University Health Science Center, Shenzhen, 518055 China; 2 Department of Thoracic Surgery, Shenzhen People’s Hospital, Shenzhen, 518020 China

**Keywords:** Non-small-cell lung cancer, Genetics research, Cancer genetics

## Abstract

Non-small cell lung cancer (NSCLC) is the leading cause of cancer mortality worldwide. The mechanisms underlying NSCLC tumorigenesis are incompletely understood. Transfer RNA (tRNA) modification is emerging as a novel regulatory mechanism for carcinogenesis. However, the role of tRNA modification in NSCLC remains obscure. In this study, HPLC/MS assay was used to quantify tRNA modification levels in NSCLC tissues and cells. tRNA-modifying enzyme genes were identified by comparative genomics and validated by qRT-PCR analysis. The biological functions of tRNA-modifying gene in NSCLC were investigated in vitro and in vivo. The mechanisms of tRNA-modifying gene in NSCLC were explored by RNA-seq, qRT-PCR, and rescue assays. The results showed that a total of 18 types of tRNA modifications and up to seven tRNA-modifying genes were significantly downregulated in NSCLC tumor tissues compared with that in normal tissues, with the 2ʹ-O-methyladenosine (Am) modification displaying the lowest level in tumor tissues. Loss- and gain-of-function assays revealed that the amount of Am in tRNAs was significantly associated with expression levels of FTSJ1, which was also downregulated in NSCLC tissues and cells. Upregulation of FTSJ1 inhibited proliferation, migration, and promoted apoptosis of NSCLC cells in vitro. Silencing of FTSJ1 resulted in the opposite effects. In vivo assay confirmed that overexpression of FTSJ1 significantly suppressed the growth of NSCLC cells. Mechanistically, overexpression of FTSJ1 led to a decreased expression of DRAM1. Whereas knockdown of FTSJ1 resulted in an increased expression of DRAM1. Furthermore, silencing of DRAM1 substantially augmented the antitumor effect of FTSJ1 on NSCLC cells. Our findings suggested an important mechanism of tRNA modifications in NSCLC and demonstrated novel roles of FTSJ1 as both tRNA Am modifier and tumor suppressor in NSCLC.

## Introduction

Lung cancer is the most common malignancy by incidence and mortality in the world^1^. Among patients with lung cancer, non-small cell lung cancer (NSCLC) accounts for ~85% of all cases^2^. Although comprehensive approaches including chemotherapy, surgery, radiotherapy, and targeted therapy have been widely used, the 5-year overall survival rate of NSCLC remains as low as 4–17%^3^. Therefore, a better understanding of the molecular mechanisms underlying NSCLC carcinogenesis is instrumental for developing novel diagnostic methods and therapies for NSCLC.

Transfer RNAs (tRNAs) are some of the most abundant molecules in a cell^4^. Traditionally, tRNAs are considered as the key component of translation, because they deliver amino acids to ribosomes and translate the mRNA information into protein sequence^5^. Recent evidence, however, suggest that tRNAs may play additional roles in a wide variety of processes, including gene expression^5^, apoptosis^6^, cell proliferation, and differentiation^7^. Studies have also indicated that dysregulation of tRNA expression is associated with human cancers, such as cervical, pancreatic, lung and breast cancers^8^. Importantly, abnormal tRNA expressions could directly control the rate of cancer protein synthesis and promote the tumorigenesis^9^. For example, dysregulation of tRNAs has been correlated to tumor proliferation and differentiation in bladder and prostate cancers, and β-cell non-Hodgkin’s lymphoma^7^. Upregulation of specific tRNAs was associated with breast cancer progression^10,11^. Nevertheless, the molecular mechanisms through which tRNA is regulated in NSCLC remain unclear.

To obtain biological functions, tRNA molecules usually undergo extensive posttranscriptional modifications during the process of maturation^12^. To date, about 90 types of tRNA modifications have been identified in *E. coli* and yeast. But tRNA modifications and their corresponding modifiers in human cells are still largely unknown^13^. In general, modifications in anticodon rings, especially at sites 34 and 37^13^, affect the accuracy and efficiency of translation; whereas insufficient modifications of D and T arms influence the stability and functional folding of tRNA structure^14^, and even lead to cell death and related diseases^15^. It is known that tRNA modifications are very conservative in evolution, suggesting that they may play important role in cells. For instance, the methylation of uracil U on the 34th nucleotide base promoted the translation speed and extension of DNA damage-related transcripts^16^. While absence of adenine 1 methylation (m^1^A) of tRNA^Lys^ induced the misfolding of tRNA, thereby affecting the normal function of tRNA^Lys 14^. Moreover, the 5C-methylcytosine (m^5^C) modification in tRNA^LEU (CAA)^ could increase the translation efficiency of ribosome response protein RPL22A^16^. However, the detailed functions and regulatory consequences remain mysterious for most tRNA modifications in humans^17^.

tRNA modifications are catalyzed by corresponding tRNA-modifying enzymes^13^. In most cases, each tRNA-modifying enzyme is only responsible for modifying particular bases at specific sites of a tRNA molecule^18^. Dysregulation of tRNA-modifying enzyme genes have been linked to several types of cancers. For example, TRMT1 catalyzed the dimethylguanosine (m^2^_2_G) modification at the 26 site of tRNA molecule, and deletion of TRMT1 inhibited cell proliferation, protein synthesis, and tolerance to oxidative stress^19^. Overexpression of NSUN2 was associated with higher rate of overall and disease progression-free survival in ovarian cancer patients, suggesting that NSUN2 was a cancer suppressor for ovarian cancer^20^. ELP3 and CTU1/2 were upregulated in human breast cancers and sustained metastasis^21^. These observations suggested that tRNA-modifying genes might play critical role in carcinogenesis. Nevertheless, the role of tRNA-modifying genes in NSCLC is still elusive.

In this study, we found that tRNA modification levels and tRNA-modifying gene expressions were generally lower in NSCLC tumor tissues than that in normal tissues. We identified that FTSJ1 expression level was associated with the amount of tRNA Am modification in NSCLC. We revealed that FTSJ1 possessed tumor suppressive capacity in vitro and in vivo. We showed that the tumor suppressive effect of FTSJ1 on NSCLC was partially mediated by DRAM1. This study provides new insights into the regulatory mechanism of tRNA modifications and biological function of FTSJ1 in NSCLC.

## Materials and methods

### Patients and tissue samples

This study was approved by the Medical Ethics Committee of Shenzhen University (Approved no. 2016002). Informed consent was obtained from each subject before sample collection. NSCLC tissues and adjacent non-tumor tissues were collected from patients with NSCLC (*n* = 7; Supplementary Table 1) in The Department of Thoracic Surgery at Shenzhen People’s Hospital in 2017. Sample size was estimated using the PS software (http://biostat.mc.vanderbilt.edu/PowerSampleSize). Assumed that *α* = 0.05 (paired *t*-test), group difference ≧0.2, and standard deviation = 0.10, 5 pairs of samples could obtain a power of 90%. Our study included seven pairs of samples, meeting the statistical rigor to interpret the data with confidence. The inclusion criteria for patients were as followings: (a) age ≧18 years; (b) pathologically confirmed diagnosis of NSCLC; (c) no previous or co-existing cancers other than NSCLC. Patients with the following characteristics were excluded: (a) had previous or co-existing cancers; (b) having received any other treatment prior to surgery; (c) had benign tumor. After surgical resection, tumor tissues and the adjacent tissues were immediately frozen in liquid nitrogen and then stored until further processing.

### tRNA isolation and HPLC-MS analysis

**Fig. 1:**
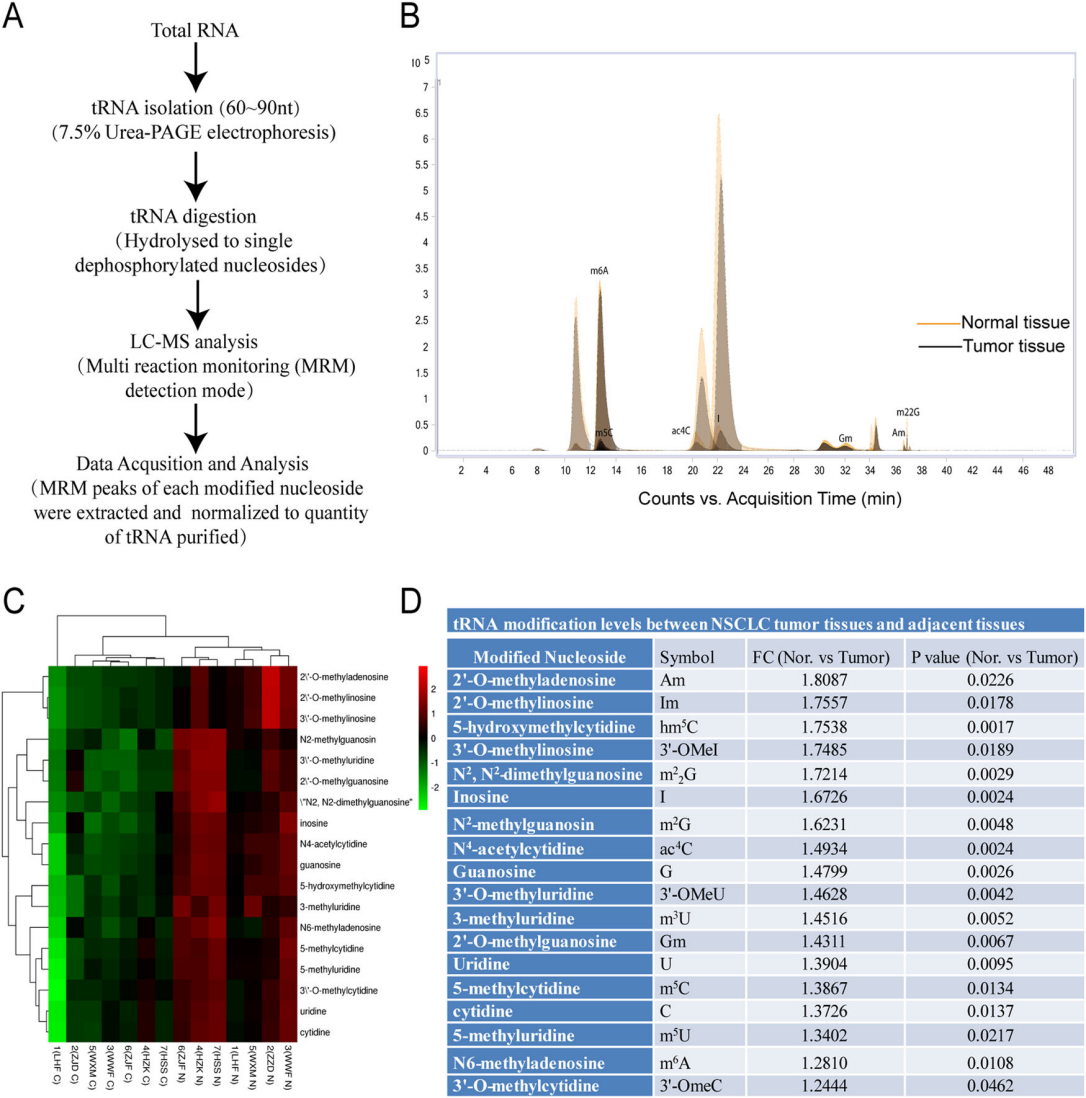
tRNA modification levels were downregulated in NSCLC tissues. **a** Procedures for the analysis of tRNA modification. **b** Representative total ion chromatogram from HPLC-MS analysis of tRNA ribonucleosides in NSCLC tumor tissues. The Y and X axes represent “intensity of the signals” and “retention time of modified nucleosides in minutes”, respectively. **c** Hierarchical cluster analysis of tRNA modification profiles in NSCLC tumor tissues and in normal tissues. Red in heat map denotes upregulation. Green refers to downregulation. **d** Differential tRNA modification levels between NSCLC tumor tissues and the adjacent tissues.

Total RNA was extracted form tissues using the Trizol reagents (Invitrogen, Shanghai, China), and the integrity and quantity of each RNA sample was examined using agarose gel electrophoresis and the NanoDrop ND-1000 spectrophotometer (Thermo Fisher Scientific, Shanghai, China). tRNA was isolated from total RNA using the 7.5% PAGE electrophoresis (Fig. 1a). Purified tRNA was hydrolyzed to single nucleosides and then dephosphorylated by enzyme mix (containing 10U Benzonase, 0.1U Phosphodiesterase I, and 1U Alkaline Phosphatase, respectively). tRNA modifications were analyzed on Agilent 6460 QQQ mass spectrometer (Agilent Technologies, Inc., CA, USA) with an Agilent 1290 HPLC system using the Multi reaction monitoring (MRM) detection mode (Fig. 1a, Supplementary Table 2). Peak information of modified nucleosides was extracted using the Agilent Qualitative Analysis software. Peak with signal-to-noise ratio no <10 was considered as a detectable nucleoside^22^. Peak areas were then normalized to the quantity of purified tRNA of each sample (Fig. 1b).

### Cell culture

NSCLC cell lines (A549, PC9, and H226) and normal human pulmonary epithelial cell line (BEAS-2B) were obtained from the Cell Bank of the Chinese Academy of Sciences (Shanghai, China). All cell lines were authenticated by matching the short-tandem repeat (STR) DNA profiles of each cell line to the corresponding standard STR in the database of ATCC and DSMZ. No contamination of mycoplasma was found in these cell lines. A549, PC9, and BEAS-2B cells were cultured in DMEM medium containing 10% fetal bovine serum (FBS, Gibco, NY, USA). H226 Cells were incubated in RPMI-1640 basic (1×) medium (Gibco, NY, USA). All cells were cultured in a humidified atmosphere with 5% CO_2_ at 37 °C.

### Plasmid construction and lentivirus production

The full-length of human FTSJ1 cDNA was synthesized by GenePharma (Suzhou, China) and cloned into the expression plasmid vector pcDNA3.1(+) (Invitrogen, Shanghai, China) that have been digested by NheI and HindIII. Lentiviral expression vector lv-FTSJ1-3xflag-ZsGreen-PURO encoding the full-length of human FTSJ1 was obtained from Hanbio Biotechnology (Shanghai, China) (Supplementary Fig. 1). To generate lentiviruses, the 293T cells were co-transfected with lentiviral vector and Lipofiter^TM^ (Hanbio Biotechnology, Shanghai, China). After 48 h of infection, lentiviral particles in the supernatant were harvested and concentrated by ultracentrifugation. All vectors were verified by sequencing and qRT-PCR assays. Lentivirus with overexpression (OE) of FTSJ1 or NC were denoted as lenti-FTSJ1 and lenti-NC, respectively.

### Cell transfection

Cells were cultured in six-well plates to a density of 70–80%, then transfection was performed using GM siRNA-mate (Genepharma, Suzhou, China) for siRNA knockdown, and Lipofectamine 3000 (Invitrogen, Shanghai, China) for plasmid transfection, respectively, following the manufacturer’s procedures. Two siRNAs (5ʹ-CCAUGAUGUUGAUGAGUAUTT-3ʹ and 5ʹ-GCAGCCGGAACUCUAGCAUTT-3ʹ) targeting human FTSJ1 mRNA sequence were synthesized by Genepharma (Suzhou, China) and were used to silence the expression of FTSJ1. A scrambled duplex RNAi oligo was used as a negative control (NC). Short hairpin RNAs (shRNAs) against FTSJ1 and NC (Genepharma, Suzhou, China) were also used for knockdown of FTSJ1. Cells were harvested after 48-h transfection.

### In vivo tumor formation assay

Four- to five-week-old female BALB/C nude mice were commercially obtained from the Guangdong Experimental Animal Center (Foshan, China). The mice were randomly divided into two groups (*n* = 6 per group) using the random number table method, and the investigator was blinded to the group allocation during the experiment. PC9 cells stably infected with lenti-FTSJ1 or empty-vector were suspended at a concentration of 2 × 10^6^ cells/100 μl. A total of 100 μl of cell suspensions were subcutaneously injected into the right flank of each nude mice. The tumor size and body weight of the mice were measured every 3 days, and tumor volumes were calculated using the formula *V* = 1/2 × *D* × *d*^2^ (*V*, volume; *D*, longitudinal diameter; *d*, latitudinal diameter). The mice were euthanized at 21 days after inoculation. Tumors were isolated and fixed in 4% formaldehyde, embedded in paraffin, and then sectioned for subsequent staining. All animals were maintained under pathogen-free conditions and experimental procedures were manipulated according to the protocols approved by the Animal Ethical Committee of Shenzhen University (Approval no. AEWC-2019009).

### RNA sequencing

To investigate the target genes regulated by FTSJ1, PC9 cells were transfected with FTSJ1 or scrambled controls for 48 h. Total RNA was isolated from PC9 cells using the TRIzol (Invitrogen, Shanghai, China) reagents, and RNA quantity and quality were measured using the NanoDrop ND-1000 Spectrophotometer (Thermo Fisher Scientific, Shanghai, China). mRNA was pulled down using the NEBNext® Poly(A) mRNA Magnetic Isolation Module (New England Biolabs, MA, USA). RNA-seq libraries were prepared using the KAPA Stranded RNA-Seq Library Prep Kit (Illumina, CA, USA), and sequencing was performed on an Illumina HiSeq 4000 by KangChen Biotech Company (Shanghai, China). Sequencing reads were trimmed using StringTie and mapped to human genome database (GRCh37) by the Hisat2 software. Differential expression (FPKM, fragments per kilobase of gene/transcript model per million mapped fragments) were calculated using the Ballgown software. The sequencing data set has been deposited in the Gene Expression Omnibus (https://www.ncbi.nlm.nih.gov/geo/) under the accession number GSE146604.

### qRT-PCR, cell proliferation, transwell, apoptosis, western blotting, and Immunohistochemistry (IHC) assays

The detailed methodology of qRT-PCR, cell proliferation, transwell, flow cytometry, western blot, and IHC assays are presented in the Supplementary materials.

### Statistical analysis

Quantitative data were presented as mean ± standard deviation (SD). Differences between two groups were assessed using Student *t*-test (two-tailed), one-way ANOVA, or the Mann–Whitney *U* test, where appropriate. Categorical variables were analyzed using the *χ*^2^ test. All statistical analyses were performed using the SAS 9.3 program (SAS Corp., NC, USA) and GraphPad Prism 7.0 (GraphPad software, Inc., USA). Data were considered to be statistically significant when *P* value (two-sided) was <0.05.

## Results

### tRNA modification levels were downregulated in NSCLC tissues

To explore the associations of tRNA modification levels with NSCLC, we compared the tRNA modification profiles in NSCLC tumor tissues with that in adjacent tissues. We used a highly accurate HPLC-MS assay to determine the modification levels of tRNAs^22,23^. The results showed that, besides native A, G, U, and C modifications, a total of 36 ribonucleoside modifications of tRNAs were detected in NSCLC tumor tissues (Supplementary Table 3). Among them, modification levels of 18 ribonucleosides (Am, Im, hm^5^C, 3ʹ-Omel, M^2^_2_G, I, M^2^G, ac^4^C, G, 3ʹ-OMeU, m^3^U, Gm, U, m^5^C, C, m^5^U, m^6^A, 3ʹ-OmeC) in tumor tissues were significantly lower than that in the adjacent tissues (all *P* values <0.05) (Fig. 1c, d), with the Am (2ʹ-O-methyladenosine) displayed lowest level in tumor tissues. These data suggesting that lower tRNA modification levels may be associated with the pathogenesis of NSCLC.

### tRNA-modifying enzyme genes were downregulated in NSCLC tumor tissues

To date, most genes encoding tRNA modification enzymes have been identified in bacteria and yeast^24,25^, and relatively little is known about tRNA-modifying genes in human cells^26^. In this study, comparative genomics approach^27^ was employed to identify potential tRNA modification enzyme genes in humans. Briefly, the protein sequences of known tRNA-modifying enzymes of *S. cerevisiae* and *E. coli* were retrieved from the NCBI database and used as query sequences. Then, BLAST was applied to map the protein sequences of yeast and bacteria to that of humans in the protein domain on pfam database (version 32.0) (http://pfam.xfam.org/). Gene homologs with an *E*-value <1.0E−5 were selected as candidate genes (Supplementary Table 4).

**Fig. 2:**
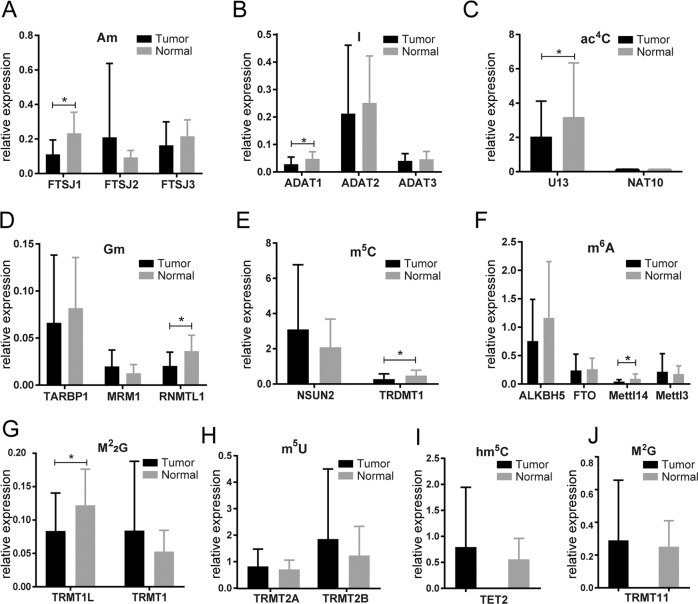
Expression levels of tRNA-modifying enzyme genes in NSCLC tumor tissues (*n* = 10) were lower than that in adjacent normal tissues (*n* = 10). Candidate tRNA modification enzyme genes in human cells were predicted using homology-based comparative genomics techniques. Expression levels of candidate tRNA-modifying genes in human NSCLC tissues were validated by qRT-PCR approach. Expression levels of tRNA-modifying genes for corresponding tRNA modifications (Am, I, ac^4^C, Gm, m^5^C, m^6^A, M^2^_2_G, m^5^U, hm^5^C, M^2^G) are showed in (**a**), (**b**), (**c**), (**d**), (**e**), (**f**), (**g**), (**h**), (**i**), and (**j**), respectively. *P < 0.05.

To validate candidate human tRNA-modifying genes identified by comparative genomics prediction, we further applied qRT-PCR assay to determine the expression levels of 23 candidate genes in an independent set of samples consisting 10 pairs of NSCLC tissues and adjacent tissues. The results showed that the expression levels of seven tRNA-modifying genes (FTSJ1, ADAT1, U13, RNMTL1, TRDMT1, METTL14, and TRMT1L) in NSCLC tumor tissues were significantly lower than that in normal tissues (all *P* values < 0.05) (Fig. 2a–j). Among these differentially expressed genes, FTSJ1, which potentially catalyzed the Am modification of tRNAs, had the smallest *P* value (*p* = 0.0124). Given that Am modification was also lowest in tumor tissues, we selected FTSJ1 for subsequent investigation.

### FTSJ1 was downregulated in NSCLC cells and associated with tRNA Am modification level

**Fig. 3:**
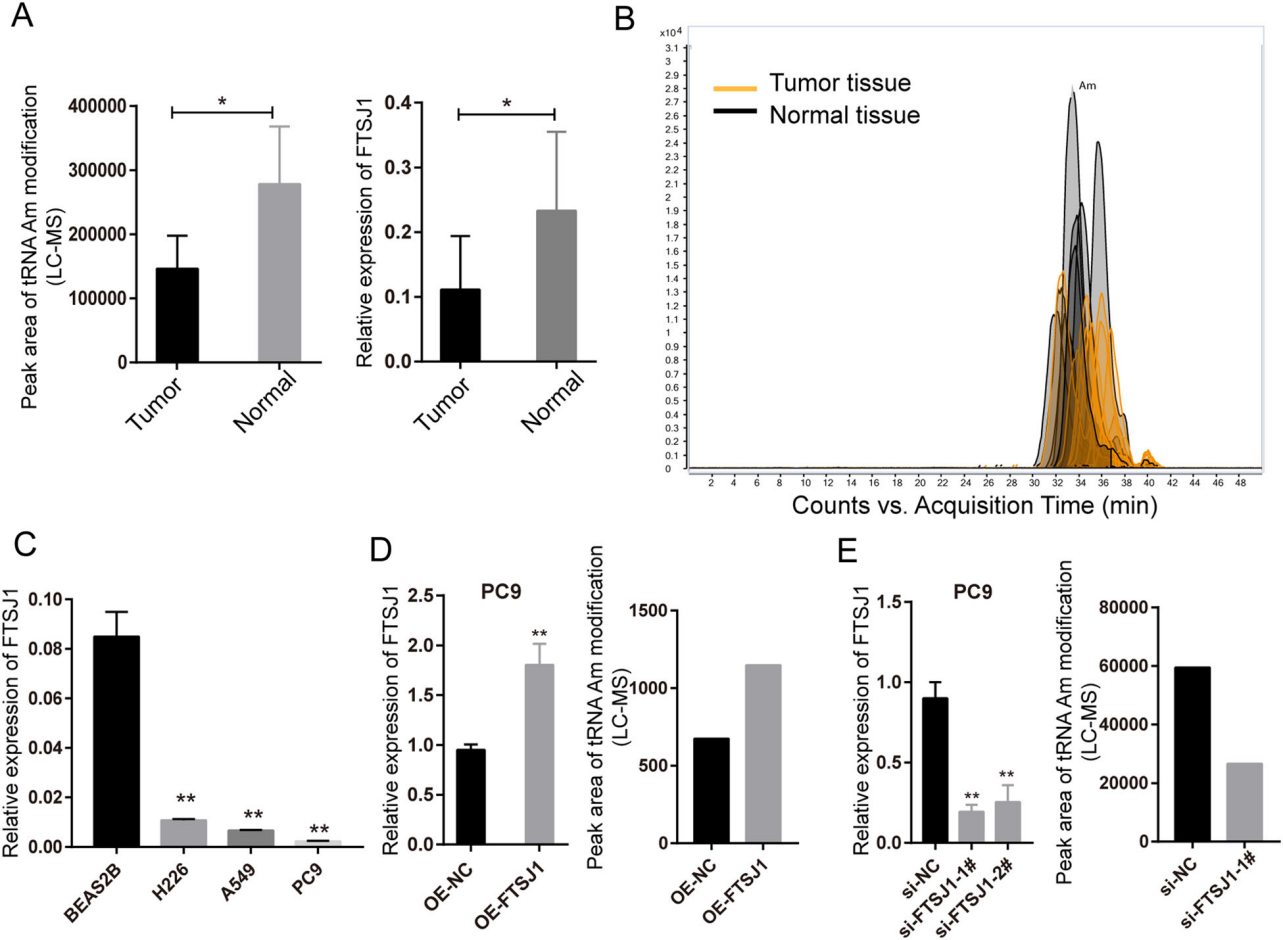
FTSJ1 expression level was associated with Am (2ʹ-O-methyladenosine) modification level of tRNAs in NSCLC. **a** Left: tRNA Am levels in NSCLC tumors were lower than that in normal tissues; right: FTSJ1 was downregulated in NSCLC tumor tissues compared with that in normal tissues. **b** Total ion chromatogram of Am detected by HPLC-MS assay. **c** Expression levels of FTSJ1 in NSCLC cells (H226, A549, and PC9) were significantly lower than that in normal bronchial epithelial cells (BEAS-2B). **d** Left: transfection of FTSJ1 into NSCLC cells resulted in increased expression of FTSJ1; right: overexpression of FTSJ1 led to increased level of tRNA Am modification in NSCLC cells. **e** Left: knockdown of FTSJ1 inhibited FTSJ1 expression in NSCLC cells; right: silencing of FTSJ1 reduced Am modification levels in NSCLC cells. *P < 0.05; **P < 0.01.

To assess the baseline levels FTSJ1 expression in NSCLC cells, qRT-PCR assay was performed on RNAs extracted from NSCLC cells (A549, PC9, and H226), and normal lung epithelial cells (BEAS-2B). Consistent with observations in NSCLC tissues (Fig. 3a, b), the expression levels of FTSJ1 in NSCLC cell lines were all significantly lower than that in BEAS-2B cells (Fig. 3c). To investigate whether FTSJ1 expression was associated with tRNA Am modification levels in NSCLC, FTSJ1, or si-FTSJ1 were transfected into PC9 cells, then expression levels of FTSJ1 and quantity of Am were determined by qRT-PCR and HPLC-MS assays. The results showed that overexpression of FTSJ1 induced increases of both FTSJ1 expression as well as tRNA Am levels in PC9 cells (Fig. 3d). In contrast, knockdown of FTSJ1 led to decreased levels of Am modification and FTSJ1 expression in PC9 cells (Fig. 3e). These data were in agreement with observations in tissues in which both tRNA Am modification levels and FTSJ1 expression were lower in NSCLC tumor tissues than in normal tissues (Fig. 3a, b). Taken together, these data indicated that FTSJ1 gene expression level was specifically related to tRNA Am modification levels in NSCLC.

### FTSJ1 suppressed proliferation, migration, and promoted apoptosis in NSLCC cells

**Fig. 4:**
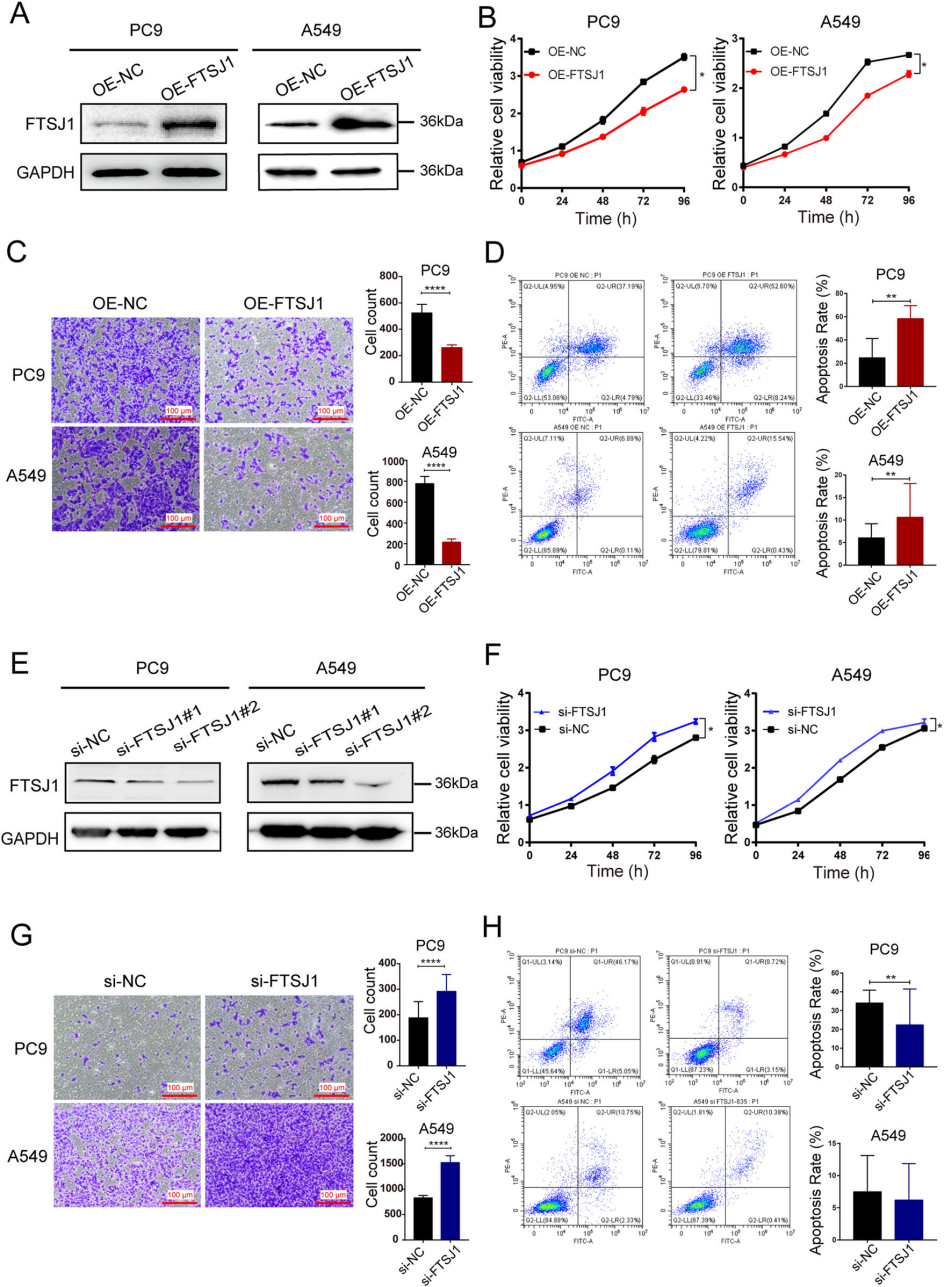
FTSJ1 suppressed the malignancy of NSCLC cells in vitro. **a** Transfection of FTSJ1 plasmids into NSCLC cells resulted in increased expression of FTSJ1 protein. **b** Overexpression of FTSJ1 inhibited cell proliferation in PC9 and A549 cells. **c** Upregulation of FTSJ1 suppressed migration rate of NSCLC cells. **d** Transfection of FTSJ1 into NSCLC cells induced increased apoptosis. **e** Validation of the suppressive efficiency of si-FTSJ1 by Western blot. **f** Knockdown of FTSJ1 promoted NSCLC cell proliferation. **g** Silencing of FTSJ1 increased migration rate of NSCLC cells. **h** si-FTSJ1 transfection repressed apoptosis in NSCLC cells. *P < 0.05; **P < 0.01; ****P < 0.0001.

To evaluate the functional roles of FTSJ1 in NSCLC, overexpression of FTSJ1 was constructed with the pcDNA3.1(+) plasmid, and the expression of FTSJ1 vector was confirmed by western blot assay in PC9 and A549 cells, respectively (Fig. 4a). It was found that overexpression of FTSJ1 significantly suppressed proliferation of both PC9 and A549 cells (Fig. 4b, Supplementary Table 5). Similarly, overexpression of FTSJ1 dramatically inhibited migration rates of A549 and PC9 cells (Fig. 4c). Furthermore, increased expression of FTSJ1 significantly promoted apoptosis rate of NSCLC cells (Fig. 4d).

To further verify the functional roles of FTSJ1 in NSCLC cells, PC9 and A549 cells were transfected with specific siRNAs or shRNA to inhibit the expression of FTSJ1 (Fig. 4e). As expected, knockdown of FTSJ1 increased proliferation rates of A549 and PC9 cells (Fig. 4f, Supplementary Table 5). Silencing of FTSJ1 also increased migration rate of A549 and PC9 cells (Fig. 4g). Compared with NC controls, knockdown of FTSJ1 induced a decreased rate of apoptotic cells in both PC9 and A549 cells (Fig. 4h). Collectively, these observations indicated that FTSJ1 may serve as an antitumor gene in NSCLC.

### Overexpression of FTSJ1 inhibited tumor growth in vivo

**Fig. 5:**
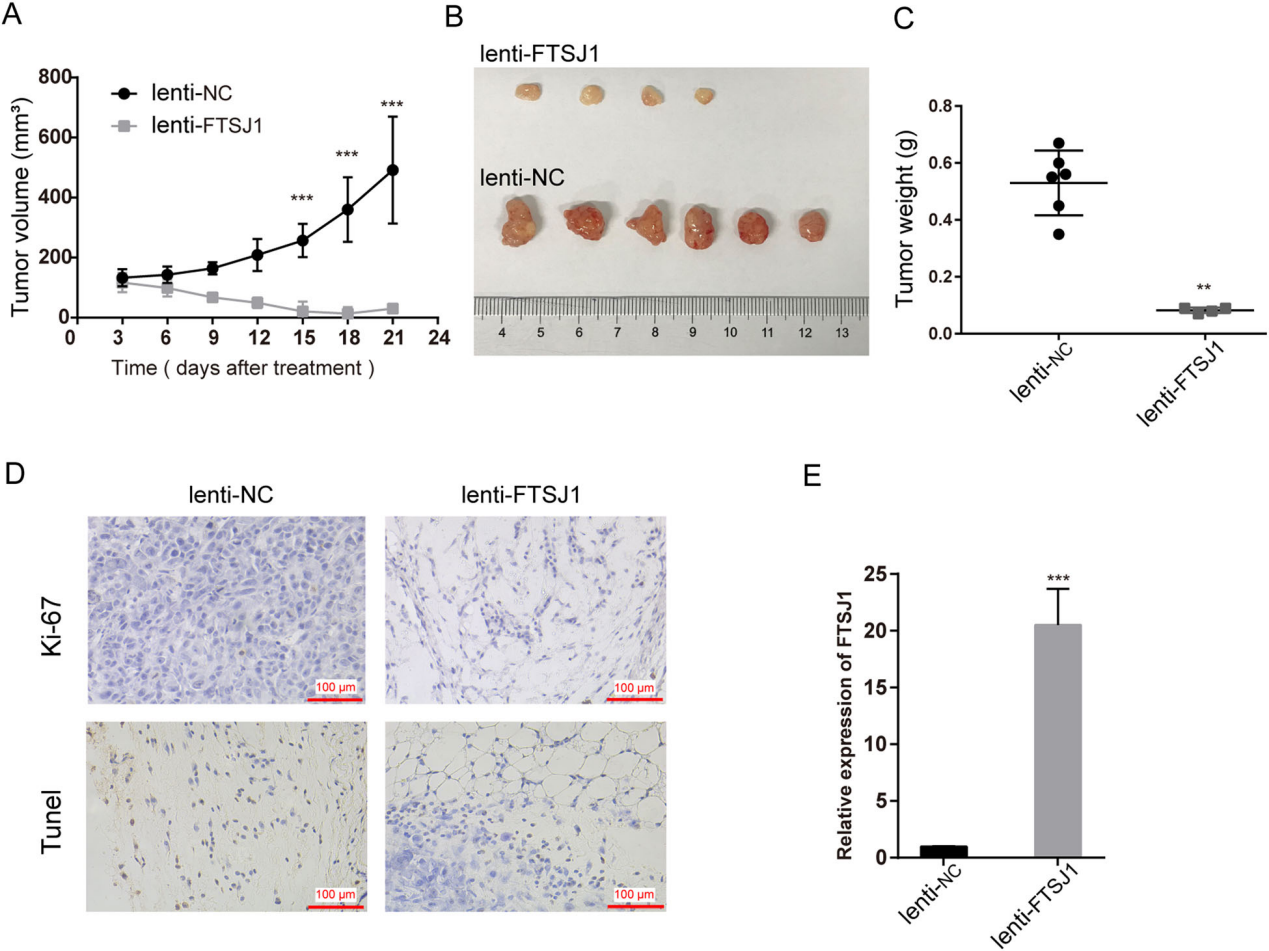
Overexpression of FTSJ1 inhibited NSCLC tumor growth in vivo. PC9 cells stably expressed FTSJ1 or NC were used for in vivo study. **a** Growth curves of tumors in nude mice injected with stably expressed FTSJ1 of PC9 cells or control cells. **b** Images of xenografts derived from PC9 cells infected with lenti-FTSJ1 (upper panel) or lenti-NC (lower panel). **c** Tumor weight of xenografts derived from PC9 cells infected with lenti-FTSJ1 or controls. **d** Tumors developed from FTSJ1-overexpression cells revealed lower Ki-76 protein and higher TUNEL staining levels than that of tumors developed by lenti-NC cells, Ki-67 immunostaining; right, TUNEL staining. **e** qRT-PCR analysis showed that the expression level of FTSJ1 in lenti-FTSJ1-treated tumors was higher than that in lenti-NC-treated xenografts. All experiments were performed in biological triplicates with three technical replicates. Data are presented as mean ± SD. *P < 0.05; **P < 0.01; ***P < 0.001.

To verify in vitro experimental results, the PC9 cell stably transfected with FTSJ1 or NC were injected into nude mice. Twenty-one days later, the growth rate, tumor size, and tumor weight in FTSJ1-overexpression group were obviously lower than those in NC group (Fig. 5a–c). IHC analysis showed that Ki-67 expression in FTSJ1-overexpression group was lower than that in NC group; whereas TUNEL staining revealed that FTSJ1-overexpression tumor tissues had more apoptotic cells that that in tumors of NC froup (Fig. 5d). Importantly, levels of FTSJ1 in tumors of FTST1-overexpression group was higher than that in NC group (*P* < 0.05; Fig. 5e). Therefore, we confirmed that FTSJ1-overexpression suppressed NSCLC in vivo and in vivo.

### FTSJ1 suppressed NSCLC cell growth partly by inhibiting DRAM1 expression

**Fig. 6:**
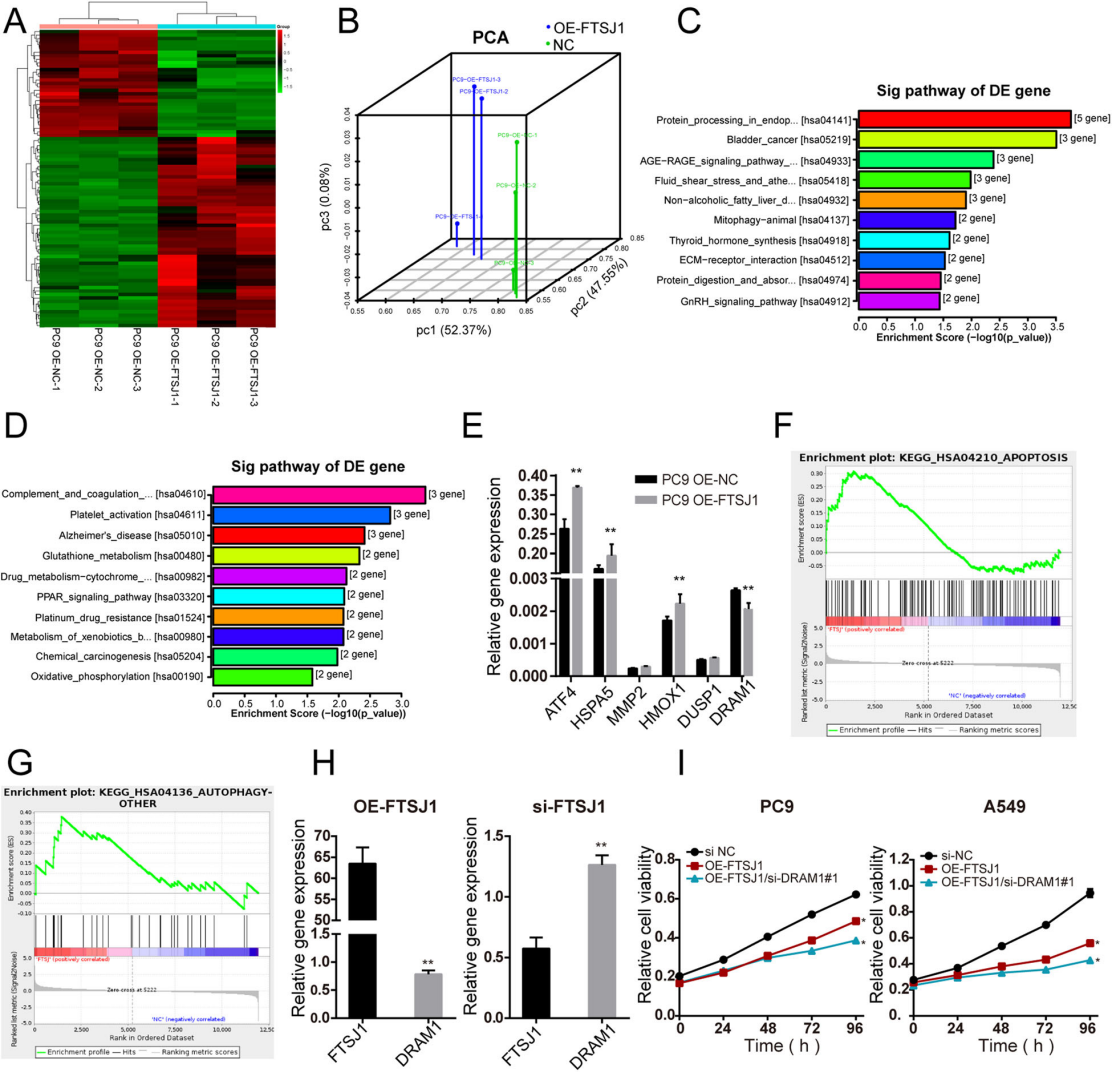
DRAM1 is a direct target gene regulated by FTSJ1. **a** Heat map generated from the RNA-seq analysis, showing differential gene expression profiles between FTSJ1-overexpression cells and control cells, with three repeats. **b** Principle component analysis (PCA) of differentially expressed genes revealed a clear separation between FTSJ-overexpression cells and control cells. **c** KEGG pathway analysis revealing that FTSJ1 activated upregulated pathways. **d** Pathway analysis showing that FTSJ1-induced downregulated genes were enriched in cancer-associated pathways. **e** Differentially expressed mRNAs regulated by FTSJ1 overexpression were verified using qRT-PCR in PC9 cells transfected with FTSJ1 or NC. **f**, **g** Gene set enrichment analysis (GSEA) of FTSJ1-overexpression cells versus control cells showed that genes were enriched in autophagy and apoptosis pathways. **h** Upregulation of FTSJ1 suppressed DRAM1 expression; knockdown of FTSJ1 promoted DRAM1 expression. **i** Rescue assay showed that si-DRAM1 substantially enhanced the inhibitiVE effect of FTSJ1 on NSCLC cell growth. *P < 0.05; **P < 0.01.

RNA-seq identified 55 upregulated and 33 downregulated (fold-change ≧ 2, *P* < 0.05) genes modified by FTSJ1 transfection (Fig. 6a, Supplementary Table 6). The principal component analysis revealed that gene expression profiles in FTSJ1-overexpression cells were distinct from that in control cells (Fig. 6b). KEGG pathway enrichment analysis suggested that upregulated genes were mainly involved in tumor-associated pathways including bladder cancer, ECM-receptor interaction, protein digestion and absorption, and GnRH signaling pathway (Fig. 6c). While downregulated genes were more enriched in metabolism-related pathways such as glutathione metabolism, PPAR signaling, platinum drug resistance, and oxidative phosphorylation (Fig. 6d). To verify target genes identified by RNA-seq, qRT-PCR assay was performed on several upregulation mRNAs. In consistent with results from that of RNA-seq, transfection of FTSJ1 into PC9 cells induced increased expression of these genes (Fig. 6e). Gene set enrichment analysis (GSEA) demonstrated that differentially expressed genes were enriched in cancer-related gene sets in glutathione metabolism, PPAR signaling, Hippo signaling, and platinum resistance (Supplementary Fig. 2).

Among those differentially expressed genes in response to FTSJ1 overexpression, we were particularly interested in DRAM1, because DRAM1 was a target gene of p53 and was involved in autophagy and apoptosis of cancer cells^28,29^. Indeed, GSEA analysis demonstrated that differential genes between FTSJ1-overexpression cells versus control cells were enriched in autophagy and apoptosis pathways (Fig. 6f, g). qRT-PCR analysis confirmed that transfection of FTSJ1 resulted in downregulation DRAM1 in NSCLC cells. On the contrary, knockdown FTSJ1 induced an increased expression of DRAM1 in NSCLC cells (Fig. 6h). Rescue assays were further performed to determine whether DRAM1 was involved in FTSJ1-mediated inhibition of NSCLC cell proliferation. PC9 and A549 cells were co-transfected with si-DRAM1 and FTSJ1 plasmid. CCK-8 assay revealed that the suppressive effects of FTSJ1 on NSCLC cell proliferation were substantially enhanced by knockdown of DRAM1 (Fig. 6i). Collectively, these findings suggested that FTSJ1 may exert an antitumor function on NSCLC in part through the downregulation of DRAM1.

## Discussion

It has been documented that tRNA modifications play critical roles in tRNA stability, translational efficiency, and fidelity^5,6^. Lack of these modifications increases the level of missense errors, codon decoding rate, and protein aggregation with deleterious consequences to the cell^13^. However, relatively little is known about the overall profiles of tRNA modification or tRNA-modifying enzyme genes in human cancers^16^. In this study, we demonstrated, for the first time, that a total of 18 types of tRNA modifications and up to 7 tRNA-modifying genes were downregulated in NSCLC tumor tissues. These findings suggested that multiple types of tRNA modifications might be involved in the pathogenesis of NSCLC.

Increasing evidence has revealed that tRNA modifications and tRNA-modifying genes are implicated in the development and progression of cancers^8,16^. Indeed, hyper-modified tRNAs and upregulation of tRNA-modifying enzymes have been found in several types of cancers^15,30^. For example, tRNA modification levels in fast-proliferating endometrial cancer cells were higher than that in normal cells^30^. ELPS and CTU1/2, the enzymes responsible for mcm^5^s^2^ modification, were upregulated in breast cancer and promoted metastasis^21^. Similarly, NSUN2 was overexpressed in breast cancer and in head and neck squamous cell carcinoma, and its expression levels were correlated with cancer development and progression^31,32^. In contrast, the TRM9L was downregulated in breast, bladder, colorectal, cervix, and testicular carcinomas; overexpression of TRM9L suppressed tumor growth in vivo^33^. In agreement with this, TRM9L expression level was also lower in ovarian cancers than that in benign tumor, and upregulation of TRM9L inhibited proliferation and promoted apoptosis in ovarian cancer cells^34^. In the present study, we found that the levels of both tRNA modification and tRNA-modifying gene expression were lower in NSCLC tumor tissues than that in normal tissues. Our findings suggested that lower tRNA modifications or decreased expression of tRNA-modifying enzyme genes may have important functions in NSCLC tumorigenesis. The mechanisms that regulate up- or downregulation of particular tRNA modifications in cancer are unclear and beyond the scope of the present study. But it is well known that tumors are characterized by increased cellular proliferation and increased protein synthesis rate, and cancer cells have distinct tRNA pools that may more or less efficiently translate subsets of oncogenes^8,13,15^. Future experiments are required to shed light on this fundamental question.

In this study, we focused on the Am modification because it was among those that displayed lowest modification levels in tumor tissues. Both comparative genomics and experimental analyses confirmed that Am levels were significantly correlated with FTSJ1 expressions. The FTSJ1 (FtsJ RNA 2ʹ-O-methyltransferase 1; Gene ID: 24140) encodes the 2′‑O‑methyltransferase that uses S‑adenosylmethionine as methyl donor^35^. Mammalian FTSJ1 is not as well characterized as its yeast homolog, but human FTSJ1 has been reported to be able to methylate the 2′-O-ribose of nucleotides at positions 32 and 34 of the tRNA anticodon loop^36^, catalyzing the formation of C^2^_2_m, C^3^_4_m, G^3^_4_m, and ncm^5^mU_34_ modifications in tRNAs^13^. Nevertheless, no reports have linked FTSJ1 expression with tRNA Am modification levels in human samples. Moreover, although deficiency of FTSJ1 has been correlated to X-linked intellectual disability and a genetically heterogeneous group of brain disorders^13,37^, the effects of FTSJ1 deficiency on other organs rather than on brain function have not been investigated. Here, we found that FTSJ1 correlated to Am modification levels and suppressed tumor growth in NSCLC. To the best of our knowledge, this is the first report showing the influence FTSJ1 on tRNA Am modification in cancer. Our findings suggested that FTSJ1-mediated tRNA Am modification might play a critical role in the pathogenesis of NSCLC.

We found that overexpression of FTSJ1 suppressed the malignant phenotypes of NSCLC in vitro and in vivo. Our findings suggested that FTSJ1 might function as a tumor suppressor in NSCLC. Translation of these findings to further validation of the antitumor effect of FTSJ1, such as the use of nanoparticles^38^ to deliver FTSJ1 to tumor in animal models, may help to development of new cancer treatment strategy. To elucidate the mechanisms of action of FTSJ1, we performed transcriptome profiling, which led to the discovery that DRAM1 expression was inhibited by FTSJ1 overexpression. We further verified that downregulation of FTSJ1 markedly increased the expression levels of DRAM1. In addition, rescue assays demonstrated that the tumor suppressive effect of FTSJ1 depended on DRAM1 expression, as co-transfection of FTSJ1 and si-DRAM1 into NSCLC cells significantly enhanced the tumor suppressive effect of FTSJ1 on cell proliferation. Collectively, multiple lines of evidence indicated that FTSJ1 may exert a tumor suppressor function partly through interacting with DRAM1 in NSCLC.

DRAM1 is a p53 target gene that codes a lysosomal membrane protein and plays a critical role in TP53-mediated autophagy and apoptosis^39^. DRAM1 was highly expressed in glioblastoma multiforme (GBM) and was associated with shorter overall survival in GBM patients, and knockdown of DRAM1 inhibited the invasion and migration capacity of glioblastoma stem cells^28^. Whereas overexpression of DRAM1 would increase p53-dependent apoptosis^29^. BAX is a pro-apoptotic protein that activates expression of both procaspase-9 and -3. It is known that DRAM1 could promote apoptosis through inhibiting BAX degradation and recruiting BAX to lysosomes through a protein–protein interaction^40^. Upregulation of DRAM1 has been associated with increased levels of irradiation-induced autophagy in breast cancer cells^41^. While downregulation of DRAM1 gene suppressed autophagy and increased chemosensitivity of acute myeloid leukemia (AML) cells^42^. In the present study, we found that FTSJ1 could interact with DRAM1 in NSCLC cells, our findings underscored the complicity of tRNA modifications and their regulatory enzymes in NSCLC. Further investigations are required to better address the underlying mechanisms between FTSJ1 and DRAM1 in NSCLC.

We recognized several limitations in this study. First, the sample size of patients with NSCLC was relatively small. Additional studies with larger populations will be necessary to confirm the contributions of tRNA modifications and tRNA-modifying genes to NSCLC. Second, although we chose to focus on DRAM1 as the target gene of FTSJ1, the interaction of FTSJ1 with other potential target genes needs to be further explored. Third, we found that FTSJ1 was homologous to TRM7, which is known to be implicated in the modification of rRNAs in yeast^43^. Whether FTSJ1 may also be able to regulate rRNA modifications in humans requires to be characterized. Fourth, the design of this study did not allow us to assess the impact of FTSJ1 on protein synthesis, future works using Ribo-Seq, Punch-P or quantitative proteomics techniques may help to define the role of FTSJ1 in translational regulation.

In summary, our current work revealed that both tRNA modifications and tRNA-modifying genes were downregulated in NSCLC. We found that FTSJ1 gene expression level was associated with the amount of Am modification in tRNAs. We showed that FTSJ1 suppressed the malignant phenotypes of NSCLC cells in vitro and in vivo. Moreover, we demonstrated that FTSJ1 exerted its suppressive role in NSCLC via interacting with DRAM1. These findings extended our knowledge regarding the functions of tRNA modifications and suggested that FTSJ1 might have therapeutic potential in the treatment of NSCLC.

## Supplementary information

Supplementary materials

Supplementary Figure 1

Supplementary Figure 2

## References

[1] Siegel RL, Miller KD, Jemal A (2018). Cancer statistics, 2018. Ca. Cancer J. Clin..

[2] Goldstraw P (2011). Non-small-cell lung cancer. Lancet.

[3] Miller KD (2016). Cancer treatment and survivorship statistics, 2016. Ca. Cancer J. Clin..

[4] Grewal SS (2015). Why should cancer biologists care about tRNAs? tRNA synthesis, mRNA translation and the control of growth. Biochim. Biophys. Acta.

[5] Raina M, Ibba M (2014). tRNAs as regulators of biological processes. Front. Genet..

[6] Lo YT, Huang HW, Huang YC, Chan JF, Hsu YH (2017). Elucidation of tRNA-cytochrome c interactions through hydrogen/deuterium exchange mass spectrometry. Biochim. Biophys. Acta.

[7] Gingold H (2014). A dual program for translation regulation in cellular proliferation and differentiation. Cell.

[8] Huang SQ (2018). The dysregulation of tRNAs and tRNA derivatives in cancer. J. Exp. Clin. Cancer Res..

[9] Khattar E (2016). Telomerase reverse transcriptase promotes cancer cell proliferation by augmenting tRNA expression. J. Clin. Invest..

[10] Krishnan P (2016). Genome-wide profiling of transfer RNAs and their role as novel prognostic markers for breast cancer. Sci. Rep..

[11] Goodarzi H (2016). Modulated expression of specific tRNAs drives gene expression and cancer progression. Cell.

[12] Frye M, Jaffrey SR, Pan T, Rechavi G, Suzuki T (2016). RNA modifications: what have we learned and where are we headed?. Nat. Rev. Genet..

[13] Pereira M (2018). Impact of tRNA modifications and tRNA-modifying enzymes on proteostasis and human disease. Int. J. Mol. Sci..

[14] Pan T (2018). Modifications and functional genomics of human transfer RNA. Cell. Res..

[15] Rapino F, Delaunay S, Zhou Z, Chariot A, Close P (2017). tRNA modification: is cancer having a wobble? Trends. Cancer.

[16] Endres L, Fasullo M, Rose R (2019). tRNA modification and cancer: potential for therapeutic prevention and intervention. Future Med. Chem..

[17] Mishima E (2014). Conformational change in transfer RNA is an early indicator of acute cellular damage. J. Am. Soc. Nephrol..

[18] Han L, Phizicky EM (2018). A rationale for tRNA modification circuits in the anticodon loop. RNA.

[19] Dewe JM, Fuller BL, Lentini JM, Kellner SM, Fu D (2017). TRMT1-catalyzed tRNA modifications are required for redox homeostasis to ensure proper cellular proliferation and oxidative stress survival. Mol. Cell. Biol..

[20] Yang JC (2017). Association of tRNA methyltransferase NSUN2/IGF-II molecular signature with ovarian cancer survival. Future Oncol..

[21] Delaunay S (2016). Elp3 links tRNA modification to IRES-dependent translation of LEF1 to sustain metastasis in breast cancer. J. Exp. Med..

[22] Su D (2014). Quantitative analysis of ribonucleoside modifications in tRNA by HPLC-coupled mass spectrometry. Nat. Protoc..

[23] Yan M (2013). A high-throughput quantitative approach reveals more small RNA modifications in mouse liver and their correlation with diabetes. Anal. Chem..

[24] El Yacoubi B, Bailly M, de Crecy-Lagard V (2012). Biosynthesis and function of posttranscriptional modifications of transfer RNAs. Annu. Rev. Genet..

[25] Jonkhout N (2017). The RNA modification landscape in human disease. RNA.

[26] de Crecy-Lagard V (2019). Matching tRNA modifications in humans to their known and predicted enzymes. Nucleic Acids Res..

[27] Wang Y (2017). Identification of tRNA nucleoside modification genes critical for stress response and development in rice and *Arabidopsis*. Bmc. Plant. Biol..

[28] Galavotti S (2013). The autophagy-associated factors DRAM1 and p62 regulate cell migration and invasion in glioblastoma stem cells. Oncogene.

[29] Takahashi M (2013). Overexpression of DRAM enhances p53-dependent apoptosis. Cancer Med..

[30] Dong C (2016). tRNA modification profiles of the fast-proliferating cancer cells. Biochem. Biophys. Res. Commun..

[31] Yi J (2017). Overexpression of NSUN2 by DNA hypomethylation is associated with metastatic progression in human breast cancer. Oncotarget.

[32] Lu L, Zhu G, Zeng H, Xu Q, Holzmann K (2018). High tRNA transferase NSUN2 gene expression is associated with poor prognosis in head and neck squamous carcinoma. Cancer Invest..

[33] Begley U (2013). A human tRNA methyltransferase 9-like protein prevents tumour growth by regulating LIN9 and HIF1-alpha. Embo. Mol. Med..

[34] Chen HM, Wang J, Zhang YF, Gao YH (2017). Ovarian cancer proliferation and apoptosis are regulated by human transfer RNA methyltransferase 9-likevia LIN9. Oncol. Lett..

[35] Guy MP, Phizicky EM (2015). Conservation of an intricate circuit for crucial modifications of the tRNAPhe anticodon loop in eukaryotes. RNA.

[36] Guy MP (2015). Defects in tRNA anticodon Loop 2’-O-methylation are implicated in nonsyndromic X-linked intellectual disability due to mutations in FTSJ1. Hum. Mutat..

[37] Jensen LR (2019). A mouse model for intellectual disability caused by mutations in the X-linked 2’Omethyltransferase Ftsj1 gene. Biochim. Biophys. Acta Mol. Basis. Dis..

[38] Li W (2019). Cold atmospheric plasma and iron oxide-based magnetic nanoparticles for synergetic lung cancer therapy. Free. Radic. Biol. Med..

[39] Crighton D (2006). DRAM, a p53-induced modulator of autophagy, is critical for apoptosis. Cell.

[40] Guan JJ (2015). DRAM1 regulates apoptosis through increasing protein levels and lysosomal localization of BAX. Cell. Death. Dis..

[41] Meng C (2018). MicroRNA-26b suppresses autophagy in breast cancer cells by targeting DRAM1 mRNA, and is downregulated by irradiation. Oncol. Lett..

[42] Li Y, Zhang G, Wu B, Yang W, Liu Z (2019). miR-199a-5p represses protective autophagy and overcomes chemoresistance by directly targeting DRAM1 in acute myeloid leukemia. J. Oncol..

[43] Chou HJ, Donnard E, Gustafsson HT, Garber M, Rando OJ (2017). Transcriptome-wide analysis of roles for tRNA modifications in translational regulation. Mol. Cell..

