# Correction: FTSJ1 regulates tRNA 2ʹ-O-methyladenosine modification and suppresses the malignancy of NSCLC via inhibiting DRAM1 expression

**DOI:** 10.1038/s41419-020-2623-9

**Published:** 2020-06-03

**Authors:** Qihan He, Lin Yang, Kaiping Gao, Peikun Ding, Qianqian Chen, Juan Xiong, Wenhan Yang, Yi Song, Liang Wang, Yejun Wang, Lijuan Ling, Weiming Wu, Jisong Yan, Peng Zou, Yuhuan Chen, Rihong Zhai

**Affiliations:** 1 School of Public Health, Guangdong Key Laboratory for Genome Stability & Disease Prevention, Carson Cancer Center, Shenzhen University Health Science Center, Shenzhen, 518055 China; 2 Department of Thoracic Surgery, Shenzhen People’s Hospital, Shenzhen, 518020 China

**Keywords:** Non-small-cell lung cancer, Genetics research, Cancer genetics

Correction to: *Cell Death and Disease*

10.1038/s41419-020-2525-x published online 11 May 2020

The original version of this article contained an error in the spelling of the author Yuchen Chen, which was incorrectly given as Yuhuan Chen. This has now been corrected in both the PDF and HTML versions of the article.

