# Supplementary material for: FTSJ1 regulates tRNA 2ʹ-O-methyladenosine modification and suppresses the malignancy of NSCLC via inhibiting DRAM1 expression
Source: Cell Death Dis. 2020 May 11;11(5):348. doi: 10.1038/s41419-020-2525-x (PMC7214438; doi:10.1038/s41419-020-2525-x)
Supplement: Supplementary file 1 — Supplementary materials [file 41419_2020_2525_MOESM1_ESM.docx]

**Supplementary materials**

**FTSJ1 regulates tRNA 2'-O-methyladenosine modification and suppresses the malignancy of NSCLC via inhibiting DRAM1 expression**

Qihan He^1*^, Lin Yang^2*^, Kaiping Gao^1*^, Peikun Ding^2^, Qianqian Chen^1^, Juan Xiong^1^, Wenhan Yang^1^, Yi Song^1^, Liang Wang^1^, Yejun Wang^1^, Lijuan Ling^2^, Weiming Wu^1^, Jisong Yan^1^, Peng Zou^1^, Yuhuan Chen^1^ and Rihong Zhai^1^

Correspondence: Rihong Zhai ([rzhai@szu.edu.cn](mailto:rzhai@szu.edu.cn))

^1^School of Public Health, Guangdong Key Laboratory for Genome Stability & Disease Prevention, Carson Cancer Center, Shenzhen University Health Science Center, Shenzhen, 518055, China

^2^Department of Thoracic Surgery, The People’s Hospital of Shenzhen, Shenzhen, 518020, China

Full list of author information is available at the end of the article.

*These authors contributed equally: Qihan He, Lin Yang, Kaiping Gao

Running title: FTSJ1 and tRNA Am modification in NSCLC

**Materials and Methods**

**qRT-PCR analysis**

Total RNA was extracted from tissues and cells using the TRIzol Reagent (Invitrogen, Shanghai, China) according to the manufacturer's protocol. RNA was reverse transcribed into complementary DNA (cDNA) using the PrimeScript™ RT reagent Kit with gDNA Eraser (Takara，Tokyo, Japan). Quantitative real time-PCR (qRT-PCR) was performed using the TB Green Primx Ex Taq (Takara，Tokyo, Japan) in a total volume of 20μl that contained 1.0μg cDNA, 10μl 20μM TB Green Premix Ex Taq II, 0.4μl of each primer and ddH_2_O. The primers used for qRT-PCR are listed in Supplementary Table 7.

**Cell proliferation assay**

Cell proliferation was measured using the CCK-8 assay. Briefly, after 48h transfection, 5×10^3^ transfected cells were digested, re-suspended, counted and placed on 96-well plates at 37 °C with 5% CO_2_ for 24 h. 10μl of CCK-8 reagent (Dojindo, Shanghai, China) was added into each well and incubated with cells for 1 h. Finally, cell viability was measured every 24 h (0, 24, 48, 72, and 96 h) and the absorbance value at 450 nm was calculated using the FLx800 Fluorescence Microplate Reader (BioTek, VT, USA). The experiments were performed at least three times for each condition.

**Transwell assay**

Cell migration ability was analyzed using the Falcon Transwell system (BD Biosciences, Shanghai, China). Transfected cells (3×10^5^) were added to the upper chamber coated with Matrigel, and then incubated with FBS-free medium. Medium containing FBS was added in the lower chamber as a chemoattractant. After incubation at 37^0^C for 24 h, cells on the surface of upper chamber were removed with a cotton swab. The cells that had invaded the bottom of the upper chamber were fixed with 10% formaldehyde for 10 min, stained with 0.1% crystal violet for 30 min, and counted under a microscope. The tests were repeated three times and an average migration rate was calculated for statistical analysis.

**Flow cytometry analysis**

Apoptosis assays were carried out using the Annexin V, FITC Apoptosis Detection Kit (Dojindo, Tokyo, Japan) according to the manufacturer’s instructions. Briefly, transfected cells were incubated at 37^0^C with 5% CO_2_ for 72 h. Then, cells were washed twice with PBS, centrifuged and suspended in binding buffer. Thereafter, cell suspension was treated with 5 µL V-fluorescein isothiocyanate (FITC)-conjugated Annexin V for 15 min in the dark at room temperature followed by the addition of 5 µL propidium iodide (PI). Lastly, cells were analyzed on the CytoFlex flow cytometer (Beckman Coulter, Inc., IN, USA) with excitation from the 488nm laser by simultaneously detect green fluorescence from the FITC-Annexin V in the 525/40nm filter and PI red fluorescence in the 585/42nm filter. CytExpert software (version 2.3) was utilized to analyze the data. Annexin V-positive and PI-negative cells were regarded as early apoptotic cells; cells displaying both Annexin V- and PI-positive represented the late apoptosis cells; dead cells were PI-positive and annexin V-negative; cells staining negative for both FITC Annexin V and PI were alive. The assays were conducted in triplicate and repeated at least 3 times.

**Western blotting**

Treated cells were lysed on ice with RIPA lysate buffer (Bethyl Laboratories, Inc. TX, USA). Cell protein lysates were separated by 10% SDS-polyacrylamide gel electrophoresis, and then transferred to polyvinylidence difluoride (PVDF) membrane (Membrane Solutions, TX, USA). The PVDF membrane was blocked with 5% non-fat milk at room temperature for 1 h and then incubated at 4^0^C with anti-FTSJ1 antibody (ab227259, Abcam, Shanghai, China) and anti-GAPDH (ab8245, Abcam, Shanghai, China) for overnight. After 1h of incubation with secondary antibody (goat anti-rabbit IgG H&L, ab6721, Abcam, Shanghai, China), the protein bands were visualized by enhanced chemiluminescence (BioRad, CA, USA) and detected with multi-function imager (Chemstudio SA2 Analytik Jena，Germany).

**Immunohistochemical (IHC) and TUNEL analysis**

Briefly, the paraffin-embedded xenograft tumors were sectioned and stained with hematoxylin and eosin (H&E), and the slides were incubated overnight with the primary antibodies, followed by incubated with the HRP-conjugated secondary antibodies for 50min. Staining was completed by incubating the samples with 3.30-diaminobenzidine, which produced a brown precipitate at the antigen site. Negative controls were prepared by omitting the primary antibodies. TUNEL (Terminal deoxynucleotidyl transferase dUTP nick end labeling) assays were conducted using a TUNEL Apoptosis Assay Kit (cat. 11684817910, Roche, Shanghai, China) according to the manufacturer’s instruction.

| **Supplementary Table 1.** Characteristics of patients with NSCLC enrolled for tRNA modification analysis | | | | |
| --- | --- | --- | --- | --- |
| **Sample ID** | **Gender** | **Age** | **TNM classification** | **Stage** |
| TM1 | Male | 68 | T2aN0M0 | IB |
| TM2 | Male | 71 | T1N0M0 | IA |
| TM3 | Male | 61 | T2bN2N0 | IIIA |
| TM4 | Female | 78 | T2aN0M0 | IB |
| TM5 | Male | 67 | T2aN0M0 | IB |
| TM6 | Female | 51 | T2aN0M0 | IB |
| TM7 | Female | 70 | T2aN2M0 | IIIA |
| TM8 | Male | 50 | T1bN0M0 | IA |

| **Supplementary Table 2.** Mass parameter used for nucleoside detection in tRNAs | |
| --- | --- |
| **Parameters** | **Values** |
| Gas temperature | 325°C |
| Gas Flow | 7 L/min |
| Nebulize | 40 psi |
| Sheath Gas Flow | 10 L/min |
| Capillary | 4000V (Positive) |
| Agilent 6460 QQQ mass spectrometer with Agilent 1260 HPLC system, SB-Aq 3.5μm 2.1x 150mm HPLC column (Agilent). | |

| **Supplementary Table 3.** Types of tRNA modifications detected by HPLC-MS in NSCLC tissues | | | | | | | | |  |
| --- | --- | --- | --- | --- | --- | --- | --- | --- | --- |
| **Number** | **Name** | **Symbol** | | **Number** | | **Name** | | **Symbol** | |
| 1 | cytidine | | C | | 21 | inosine | I | |  |
| 2 | adenosine | | A | | 22 | 2′-O-methylguanosine | Gm | |  |
| 3 | guanosine | | G | | 23 | 1-methylguanosine | m^1^G | |  |
| 4 | uridine | | U | | 24 | 7-methylguanosine | m^7^G | |  |
| 5 | 3′-O-methyladenosine | | 3'-OMeA | | 25 | N^2^-methylguanosin | m^2^G | |  |
| 6 | 2′-O-methylcytidine | | Cm | | 26 | 3′-O-methylinosine | 3'-OMeI | |  |
| 7 | 3-methylcytidine | | m^3^C | | 27 | 2-thiouridine | s^2^U | |  |
| 8 | 5-methylcytidine | | m^5^C | | 28 | 4-thiouridine | s^4^U | |  |
| 9 | N^6^-isopentenyladenosine | | i6A | | 29 | 5-methyluridine | m^5^U | |  |
| 10 | 5,2′-O-dimethylcytidine | | m^5^Cm | | 30 | N^4^-acetylcytidine | ac^4^C | |  |
| 11 | 1-methyladenosine | | m^1^A | | 31 | 3′-O-methyluridine | 3'-OMeU | |  |
| 12 | 2-thiocytidine | | s^2^C | | 32 | 5-methyl-2-thiouridine | m^5^s^2^U | |  |
| 13 | N^2^, N^2^, 7-trimethylguanosine | | m^2,2,7^G | | 33 | 5-methoxyuridine | mo5U | |  |
| 14 | N4-acetyl-2′-O-methylcytidine | | ac^4^Cm | | 34 | pseudouridine | Ψ | |  |
| 15 | N^6^-methyladenosine | | m^6^A | | 35 | 2′-O-methylinosine | Im | |  |
| 16 | 3′-O-methylcytidine | | 3'-OmeC | | 36 | 3-methyluridine | m^3^U | |  |
| 17 | 2′-O-methyladenosine | | Am | | 37 | 3′-O-methylguanosine | 3'-OMeU | |  |
| 18 | N^2^ ,N^2^-dimethylguanosine | | m^2^_2_G | | 38 | 1-methylpseudouridine | m^1^Ψ | |  |
| 19 | 5′-O-methylthymidine | | 5'-OMeT | | 39 | 5-hydroxymethylcytidine | hm^5^C | |  |
| 20 | 2′-O-methyluridine | | Um | | 40 | 5,2′-O-dimethyluridine | m^5^Um | |  |

| **Supplementary Table 4.** Human tRNA modification enzyme genes identified by homology–based comparative genomics techniques | | | | | | | | | |
| --- | --- | --- | --- | --- | --- | --- | --- | --- | --- |
| **Reference** | **Human-protein** | **Human-Gene** | | **Identities** | | **# Matched_AA** | **E-value** | | **Score** |
| TMCA_AC4C | NP_001137502.1 | NAT10 | | 28.626 | | 262 | 2.09E-16 | | 85.5 |
| TMCA_AC4C | NP_078938.2 | NAT10 | | 28.244 | | 262 | 2.35E-16 | | 85.1 |
| TADA_I | NP_872309.2 | ADAT2 | | 34.437 | | 151 | 9.26E-24 | | 94 |
| TADA_I | NP_001273188.1 | ADAT2 | | 30.952 | | 126 | 5.08E-14 | | 67.4 |
| TADA_I | NP_001316462.1 | ADAT3 | | 44.118 | | 68 | 3.41E-11 | | 62.4 |
| TADA_I | NP_612431.2 | ADAT3 | | 44.118 | | 68 | 4.01E-11 | | 62 |
| TRMA_M5U | NP_892029.2 | TRMT2A | | 29.012 | | 162 | 5.32E-10 | | 62.8 |
| TRMA_M5U | NP_001244923.1 | TRMT2A | | 30.894 | | 123 | 7.85E-09 | | 58.9 |
| TRMA_M5U | NP_001317968.1 | TRMT2A | | 26.111 | | 180 | 1.67E-06 | | 51.6 |
| TAD2_I | NP_872309.2 | ADAT2 | | 39.286 | | 168 | 2.11E-34 | | 123 |
| TAD2_I | NP_001273188.1 | ADAT2 | | 40.146 | | 137 | 2.55E-27 | | 103 |
| TAD3_I | NP_612431.2 | ADAT3 | | 24.911 | | 281 | 8.05E-13 | | 70.1 |
| TAD3_I | NP_001316462.1 | ADAT3 | | 24.911 | | 281 | 8.46E-13 | | 70.1 |
| TRM1_M22G | NP_001338690.1 | TRMT1 | | 37.205 | | 551 | 1.79E-113 | | 352 |
| TRM1_M22G | NP_060192.1 | TRMT1 | | 37.205 | | 551 | 6.12E-113 | | 352 |
| TRM1_M22G | NP_001136026.1 | TRMT1 | | 35.273 | | 550 | 4.43E-102 | | 323 |
| TRM1_M22G | NP_001338691.1 | TRMT1 | | 36.364 | | 330 | 7.71E-56 | | 194 |
| TRM11_M2G | NP_001026882.2 | TRMT11 | | 34.216 | | 453 | 4.11E-84 | | 266 |
| TRM11_M2G | NP_001337509.1 | TRMT11 | | 33.407 | | 452 | 5.50E-80 | | 255 |
| TRM11_M2G | NP_001337513.1 | TRMT11 | | 34.091 | | 396 | 1.12E-72 | | 235 |
| TRM11_M2G | NP_001337514.1 | TRMT11 | | 34.63 | | 257 | 4.26E-42 | | 151 |
| TRM11_M2G | NP_001337517.1 | TRMT11 | | 32.99 | | 194 | 3.13E-26 | | 107 |
| TRM112_M2G | NP_001273011.1 | TRMT112 | | 29.231 | | 130 | 2.36E-15 | | 68.9 |
| TRM112_M2G | NP_057488.1 | TRMT112 | | 30 | | 130 | 2.77E-15 | | 68.9 |
| TRM112_M2G | NP_001273013.1 | TRMT112 | | 27.184 | | 103 | 9.09E-06 | | 43.5 |
| TRM3_GM | NP_005637.3 | TARBP1 | | 37.719 | | 228 | 2.58E-38 | | 159 |
| TRM7_GM | NP_036412.1 | FTSJ1 | | 48.571 | | 315 | 1.06E-87 | | 267 |
| TRM7_GM | NP_803188.1 | FTSJ1 | | 48.254 | | 315 | 2.37E-87 | | 266 |
| TRM7_GM | NP_060117.3 | FTSJ3 | | 43.062 | | 209 | 7.49E-44 | | 161 |
| TRM7_GM | NP_037525.1 | MRM2 | | 36.585 | | 205 | 2.23E-31 | | 119 |
| TRM7_GM | NP_001269086.1 | FTSJ1 | | 42.012 | | 169 | 7.04E-29 | | 111 |
| TRM4_M5C | NP_060225.4 | NSUN2 | | 36.034 | | 716 | 6.32E-145 | | 442 |
| TRM4_M5C | NP_001180384.1 | NSUN2 | | 34.358 | | 716 | 1.68E-128 | | 398 |
| TRM4_M5C | NP_001245239.1 | NOP2 | | 26.923 | | 416 | 2.17E-27 | | 119 |
| TRM4_M5C | NP_006161.2 | NOP2 | | 27.053 | | 414 | 5.33E-27 | | 119 |
| TRM4_M5C | NP_001245237.1 | NOP2 | | 27.053 | | 414 | 5.46E-27 | | 119 |
| TRM4_M5C | NP_001245238.1 | NOP2 | | 27.053 | | 414 | 9.83E-27 | | 118 |
| TRM4_M5C | NP_001338047.1 | NSUN6 | | 30.409 | | 171 | 2.72E-14 | | 76.3 |
| TRM4_M5C | NP_001161820.1 | NSUN5 | | 29.412 | | 272 | 3.87E-14 | | 76.6 |
| TRM4_M5C | NP_683759.1 | NSUN5 | | 29.412 | | 272 | 3.87E-14 | | 77 |
| TRM4_M5C | NP_001161819.1 | NSUN5 | | 29.412 | | 272 | 4.91E-14 | | 76.6 |
| TRM4_M5C | NP_060514.1 | NSUN5 | | 29.412 | | 272 | 6.08E-14 | | 76.3 |
| TRM4_M5C | NP_001338046.1 | NSUN6 | | 30.409 | | 171 | 8.74E-14 | | 75.9 |
| TRM4_M5C | NP_071355.1 | NSUN3 | | 25 | | 240 | 1.12E-13 | | 74.7 |
| TRM4_M5C | NP_001338044.1 | NSUN6 | | 30.409 | | 171 | 1.56E-13 | | 75.1 |
| TRM4_M5C | NP_872349.1 | NSUN6 | | 30.409 | | 171 | 1.58E-13 | | 75.1 |
| TRM4_M5C | NP_001338045.1 | NSUN6 | | 30.409 | | 171 | 2.22E-13 | | 74.7 |
| TRM4_M5C | NP_001243056.1 | NSUN4 | | 30.556 | | 180 | 2.20E-12 | | 70.5 |
| TRM4_M5C | NP_950245.2 | NSUN4 | | 30.556 | | 180 | 2.71E-12 | | 70.9 |
| TRM2_M5U | NP_892029.2 | TRMT2A | | 27.805 | | 205 | 3.45E-17 | | 87 |
| TRM2_M5U | NP_001317968.1 | TRMT2A | | 26.009 | | 223 | 1.02E-14 | | 79.3 |
| TRM2_M5U | NP_001244923.1 | TRMT2A | | 30.323 | | 155 | 2.55E-14 | | 77.8 |
| TRM2_M5U | NP_079193.2 | TRMT2B | | 23.457 | | 162 | 5.70E-09 | | 60.8 |
| TRM2_M5U | NP_001161443.1 | TRMT2B | | 23.457 | | 162 | 6.09E-09 | | 60.5 |
| TRM13_AM | NP_061956.2 | TRMT13 | | 28.516 | | 519 | 2.80E-42 | | 158 |
| \| **Supplementary Table 5.** Proliferation rate of NSCLC cells regulated by over-expression of FTSJ1 or si-FTSJ1 \| \| \| \| \| \| \| \| \| \| \| \| \| \| --- \| --- \| --- \| --- \| --- \| --- \| --- \| --- \| --- \| --- \| --- \| --- \| --- \| \| Time (h) \| Mean OD \| \| SD \|  \| Mean OD \| \| SD \| \| *Growth rate (%) \| \| P-value \| \| \|  \| **A549 (OE-NC)** \| \| \|  \| **A549 (OE-FTSJ1)** \| \| \|  \| \| \|  \| \| \| 0 \| 0.437 \| 0.001 \| \|  \| 0.401 \| 0.002 \| \| -8.38% \| \| \| 0.0386 \| \| \| 24 \| 0.820 \| 0.006 \| \|  \| 0.666 \| 0.010 \| \| -18.81% \| \| \|  \| \| \| 48 \| 1.487 \| 0.006 \| \|  \| 0.994 \| 0.012 \| \| -33.12% \| \| \|  \| \| \| 72 \| 2.525 \| 0.047 \| \|  \| 1.849 \| 0.036 \| \| -26.76% \| \| \|  \| \| \| 96 \| 2.668 \| 0.022 \| \|  \| 2.283 \| 0.052 \| \| -14.42% \| \| \|  \| \| \|  \|  \|  \| \|  \|  \|  \| \|  \| \| \| \|  \| \|  \| **PC9 (OE-NC)** \| \| \|  \| **PC9 (OE-FTSJ1)** \| \| \|  \| \|  \| \| \| \| 0 \| 0.688 \| 0.014 \| \|  \| 0.599 \| 0.007 \| \| -13.03% \| \| 0.0375 \| \| \| \| 24 \| 1.112 \| 0.011 \| \|  \| 0.919 \| 0.014 \| \| -17.36% \| \|  \| \| \| \| 48 \| 1.814 \| 0.074 \| \|  \| 1.370 \| 0.023 \| \| -24.49% \| \|  \| \| \| \| 72 \| 2.844 \| 0.039 \| \|  \| 2.050 \| 0.077 \| \| -27.93% \| \|  \| \| \| \| 96 \| 3.508 \| 0.064 \| \|  \| 2.638 \| 0.043 \| \| -24.81% \| \|  \| \| \| \|  \|  \|  \| \|  \|  \|  \| \|  \| \|  \| \| \| \|  \| **A549 (si-NC)** \| \| \|  \| **A549 (si-FTSJ1)** \| \| \|  \| \|  \| \| \| \| 0 \| 0.467 \| 0.001 \| \|  \| 0.516 \| 0.010 \| \| +10.49% \| \| 0.0280 \| \| \| \| 24 \| 0.840 \| 0.011 \| \|  \| 1.145 \| 0.016 \| \| +36.30% \| \|  \| \| \| \| 48 \| 1.687 \| 0.030 \| \|  \| 2.210 \| 0.007 \| \| +30.96% \| \|  \| \| \| \| 72 \| 2.553 \| 0.029 \| \|  \| 2.994 \| 0.033 \| \| +17.25% \| \|  \| \| \| \| 96 \| 3.063 \| 0.041 \| \|  \| 3.219 \| 0.080 \| \| +5.09% \| \|  \| \| \| \|  \|  \|  \| \|  \|  \|  \| \|  \| \|  \| \| \| \|  \| **PC9 (si-NC)** \| \| \|  \| **PC9 (si-FTSJ1)** \| \| \|  \| \|  \| \| \| \| 0 \| 0.616 \| 0.011 \| \|  \| 0.719 \| 0.021 \| \| +16.73% \| \| 0.0174 \| \| \| \| 24 \| 0.972 \| 0.011 \| \|  \| 1.168 \| 0.003 \| \| +20.20% \| \|  \| \| \| \| 48 \| 1.461 \| 0.027 \| \|  \| 1.913 \| 0.086 \| \| +30.94% \| \|  \| \| \| \| 72 \| 2.218 \| 0.080 \| \|  \| 2.826 \| 0.088 \| \| +27.40% \| \|  \| \| \| \| 96 \| 2.804 \| 0.023 \| \|  \| 3.239 \| 0.052 \| \| +15.49% \| \|  \| \| \| \| * Compared with that of NC \| \| \| \| \| \| \| \| \| \| \| \| \|   **Supplementary Table 6.** Top differentially expressed genes regulated by FTSJ1 overexpression | | | | | | | | | |
| **Track_id** | | | **Gene_Name** | | ***Fold_Change** | | | **P_value** | |
| ENSG00000068438.14_2 | | | FTSJ1 | | 49.16 | | | 2.01E-06 | |
| ENSG00000196611.4_2 | | | MMP1 | | 2.89 | | | 1.69E-05 | |
| ENSG00000185955.4_3 | | | C7orf61 | | 2.67 | | | 0.0013 | |
| ENSG00000164692.17_2 | | | COL1A2 | | 2.26 | | | 0.0092 | |
| ENSG00000189252.4_2 | | | SPANXN3 | | 2.12 | | | 0.0002 | |
| ENSG00000108821.13_3 | | | COL1A1 | | 2.09 | | | 0.0025 | |
| ENSG00000160446.18_3 | | | ZDHHC12 | | 2.05 | | | 0.0115 | |
| ENSG00000087074.7_3 | | | PPP1R15A | | 2.00 | | | 0.0079 | |
| ENSG00000118137.9_2 | | | APOA1 | | -5.00 | | | 1.31E-05 | |
| ENSG00000151967.18_3 | | | SCHIP1 | | -2.50 | | | 0.0001 | |
| ENSG00000243955.5_2 | | | GSTA1 | | -2.17 | | | 0.0002 | |
| ENSG00000103404.14_3 | | | USP31 | | -2.13 | | | 0.0008 | |
| ENSG00000110245.11_2 | | | APOC3 | | -2.08 | | | 0.0004 | |
| ENSG00000136048.13_3 | | | DRAM1 | | -2.04 | | | 0.0036 | |
| ENSG00000145192.12_2 | | | AHSG | | -2.03 | | | 0.00019 | |
| ENSG00000164144.15_3 | | | ARFIP1 | | -2.00 | | | 0.0001 | |
| * Compared with gene expression levels in control cells. | | | | | | | | | |

| **Supplementary Table 7.** Primer sequences used for qRT-PCR analysis |
| --- |
| **Primers Sequences** |
| FTSJ1_qPCR _F CCATTCTTACGACCCAGATTTCA  FTSJ1_qPCR _R CCCTCTAGGTCCAGTGGGTAAC  NAT10_qPCR _F CTGAGAATAAGACCACGACGACA  NAT10_qPCR _R GCAATCCAGGCACAGCAAGT  U13_qPCR _F TGGGTGTTCATACGCTTGTG  U13_qPCR _R AGACGGGTAATGTGCCCACG  ADAT1_qPCR _F GTTTGACGCTCTGCAAGTTTTAC  ADAT1_qPCR _R CCTGATACCTCTGAGAACAAGTCC  ADAT2_qPCR _F TTGGTGTCGTCAAAGTGGC  ADAT2_qPCR _R TTCATCAGGCGGAGAGCAG  ADAT3_qPCR _F CAAGCCTGACCGTGGATTT  ADAT3_qPCR _R GGGCTGTTTATTTTCCCATCA  TRMT1_qPCR _F CTCTCCCACGCCTGTAAGAAC  TRMT1_qPCR _R CTCCCAGCAACGCATGATG  TRMT1L_qPCR _F TCTCAAGCAGGCTTTCGAGTA  TRMT1L _qPCR _R TGTATCTTCAGATGCTGACTGGAC  TRMT11_qPCR _F GTTCACAGAAGGAGATACCAAAGG  TRMT11_qPCR _R CAGATGATAACTCAAGGAAACAGGA  TARBP1_qPCR _F GTGGTCTGAGAGGAAAAAAGATGAG  TARBP1_qPCR _R CTCTGACACCGCATATTCAAACA  MRM1_qPCR _F CTGGACACAATGTGCCGCTAC  MRM1_qPCR _R CCTTATCCACTCCGAGGAAGTGT  RNMTL1_qPCR _F ATATCCAAAGACTCAGCTTCAGCA  RNMTL1_qPCR _R CAGCTGCAGATCTCAGAATTGTC  ALKBH5_qPCR _F CTTCGCAGTGTTCTAAACAAAGTTC  ALKBH5_qPCR _R AGAAGTGGAGAACAGCAGAGCCT  FTO_qPCR _F CATGGGCTTAAGCAAGAGCAGT  FTO_qPCR _R GAAGGTCCTGTTTAACACCAAATCA  METTL14_qPCR _F CTCTGTTCGTAAGCTCCCGGT  METTL14_qPCR _R GGCTTTTCACTATCCCGAGTACTT  METTL3_qPCR _F CCACTCAAGATGGGGTAGAAAG  METTL3_qPCR _R GCTTGGAATGGTCAGCATAGG  NSUN2_qPCR _F AGCATACCCAGGCTCCAGATAG  NSUN2_qPCR _R CTGCAAGCTATTTAAGGTGGTCC  TRDMT1_qPCR _F TGCGGGTGCTGGAGCTATAC  TRDMT1_qPCR _R CTTCATTAGCGACAGTGTTGACATC  TRMT2A_qPCR _F CTAACCGGGTGAAGGGCATT  TRMT2A _qPCR _R CCTCTCAAACAGGATGAGCATCT  TRMT2B _qPCR _F TATTCCTTCGACAGTCTCCATTG  TRMT2B _qPCR _R GGAAAGTGATGATAGCCATTGTG  FTSJ2_qPCR _F CAGCTCTCCTGTTGGCTTCGT  FTSJ2_qPCR _R CCATGTCGCTCAGAATCACATC  FTSJ3_qPCR _F TGCCAAGTTTATGCCTGTATCC  FTSJ3_qPCR _R CCCATCATTGAGCACAACATC  TET1_qPCR _F TAATTCTGAGCCTTCCACTGGT  TET1_qPCR _R CATTGGAGAAGAGGCAAGGTC  TET2_qPCR _F GAACACCTCAAGCATAACCCAC  TET2_qPCR _R TTGTCTCGACCCTTCAGAATCTC  TET3_qPCR _F GCGAAAATGTGAGGTGCTGA  TET3_qPCR _R ACTGGCCCTGAGTCCATCTG  DRAM1_qPCR _F AGTGCTTGGATTGGTGGGATG  DRAM1_qPCR _R GATGGACTGTAGGAGCGTGTA  ATF4_qPCR _F TCCTGTCCTCCACTCCAGATCA  ATF4_qPCR _R CTGGGCTCATACAGATGCCACT  DDIT3_qPCR _F GAACGGCTCAAGCAGGAAATC  DDIT3_qPCR _R TGGGTAGTGTGGCCCAAGTG  HSPA5_qPCR _F ATCACAATCACCAATGACCAGAATC  HSPA5_qPCR _R TGTCTTCCTCAGCAAACTTCTCAG  PPP1R15A_qPCR _F CGAGTGGCCATCTATGTACCTG  PPP1R15A _qPCR _R TGGACAGTGACCTTCTCGGA  XBP1_qPCR _F GGAATGAAGTGAGGCCAGTGG  XBP1_qPCR _R ATACCGCCAGAATCCATGGG  MMP1_qPCR _F CGCACAAATCCCTTCTACCC  MMP1_qPCR _R CCGGACTTCATCTCTGTCGG  MMP2_qPCR _F GCCCAAGAATAGATGCTGACTG  MMP2_qPCR _R GGCTGCGTTGAAAATATCAAAG  RASSF1_qPCR _F AGGGAGTGCTGGCTCACAGTAC  RASSF1_qPCR _R GGCTTTGATTAGCAAATAAGGTATG  HMOX1_qPCR _F GGGAATTCTCTTGGCTGGCTT  HMOX1_qPCR _R CCTGGATGTGCTTTTCGTTGG  COL1A2_qPCR _F AAAACATCCCAGCCAAGAACT  COL1A2_qPCR _R CTGCCAGCATTGATAGTTTCTC  DUSP1_qPCR _F CGCTCCTCTCTCAGTCCAAAAG  DUSP1_qPCR _R GCGGTGCTCTTTGTCTGTTCT  COL1A1_qPCR _F CAAGGTGTTGTGCGATGACG  COL1A1_qPCR _R TTGGTCGGTGGGTGACTCTG  CITED2_qPCR _F TCGACGAGGAAGTTCTTATGTCCT  CITED2_qPCR _R TTTCGATCGAGTCAACAGCTCAC  FTSJ1-si-835 sense（5'-3'） CCAUGAUGUUGAUGAGUAUTT  FTSJ1-si-835 antisense（5'-3'） AUACUCAUCAACAUCAUGGTT  FTSJ1-si-1014 sense（5'-3'） GCAGCCGGAACUCUAGCAUTT  FTSJ1-si-1014 antisense（5'-3'） AUGCUAGAGUUCCGGCUGCTT |

**Supplementary Figure and Table legends**

**Supplementary Table 1** Characteristics of patients with NSCLC enrolled for tRNA modification analysis.

**Supplementary Table 2** Mass parameter used for nucleoside detection in tRNAs.

**Supplementary Table 3** Types of tRNA modifications detected by HPLC-MS in NSCLC tissues.

**Supplementary Table 4** Human tRNA modification enzyme genes identified by homology–based comparative genomics techniques.

**Supplementary Table 5** Proliferation rate of NSCLC cells regulated by over-expression of FTSJ1 or si-FTSJ1.

**Supplementary Table 6** Top differentially expressed genes regulated by FTSJ1 overexpression.

**Supplementary Table 7** Primer sequences used for qRT-PCR analysis.

**Supplementary Fig. 1** Full sequence of FTSJ1. **a** Vector for construction of FTSJ1-overexpression plasmid**. b** Full sequence of FTSJ1**.**

**Supplementary Fig. 2** Gene set enrichment analysis (GSEA) of FTSJ1-overexpression cells versus control cells. Gene were enriched in **a** glutathione metabolism **b** PPAR signaling **c** HIPPO signaling, and **d** platinum resistance pathways， respectively**.**
