# Supplementary figures and images for: FTSJ1 regulates tRNA 2ʹ-O-methyladenosine modification and suppresses the malignancy of NSCLC via inhibiting DRAM1 expression
Source: Cell Death Dis. 2020 May 11;11(5):348. doi: 10.1038/s41419-020-2525-x (PMC7214438; doi:10.1038/s41419-020-2525-x)

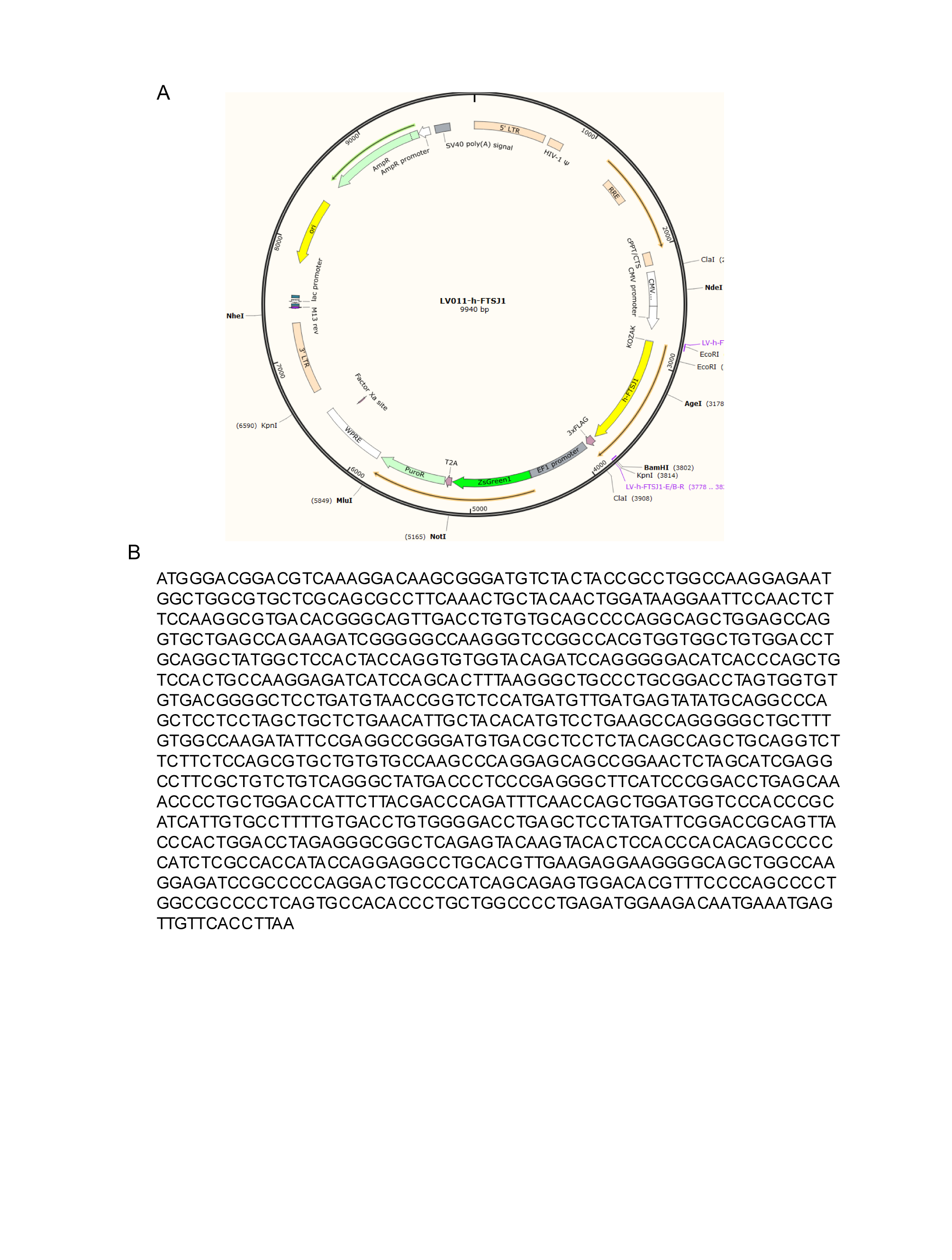

Supplement: Supplementary file 2 — Supplementary Figure 1 [file 41419_2020_2525_MOESM2_ESM.tif]

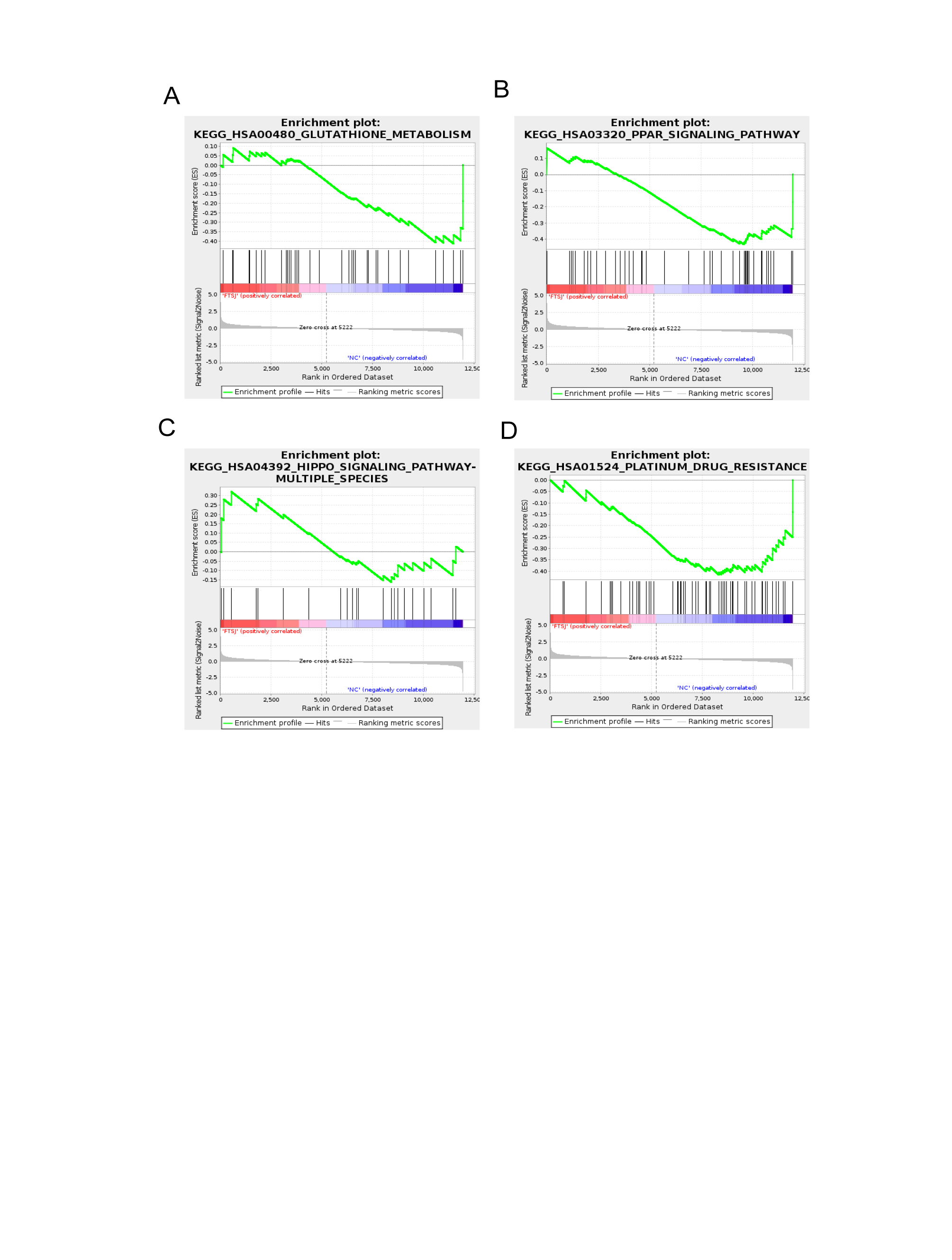

Supplement: Supplementary file 3 — Supplementary Figure 2 [file 41419_2020_2525_MOESM3_ESM.tif]
